# The Relative Age Effect in Spanish Female Soccer Players. Influence of the Competitive Level and a Playing Position

**DOI:** 10.1515/hukin-2015-0041

**Published:** 2015-07-10

**Authors:** Silvia Sedano, Roel Vaeyens, Juan Carlos Redondo

**Affiliations:** 1 Laboratory of Physiology. European University Miguel de Cervantes, Valladolid, Spain.; 2 Ghent University, Department of Movement and Sports Sciences, Belgium.; 3 Faculty of Sports Sciences, Department of Movement and Sport Sciences, University of León, Spain.

**Keywords:** birth-date, talent selection, biological maturity, female sport

## Abstract

The purposes of the study were to examine relative age effects (RAEs) in Spanish female soccer and to identify the influence of a playing position. The sample comprised all female players (n=4035) of five different competitive levels in the 2010–2013 seasons: First, Second and Third divisions (n=936, n=1711 and n=870, respectively), and National and Regional (n=232 and n=286, respectively) teams were included. Differences between the observed and expected birth-date distributions were tested based on data from the general Spanish population, using the chi-square statistic followed up by calculating odds ratios and 95% confidence intervals. Results revealed that the birth-date distributions of almost all groups of football players showed an overrepresentation of players born in the first quartile. Only in the lowest level was age distribution not significantly different from that of the general population. Moreover, the RAE risk progressively increased with a higher level of involvement. It was also observed that at some playing positions the birth-date distributions were significantly biased. That was the case for goalkeepers and defenders. It could be concluded that in the current structure of Spanish female soccer there is a relative age effect, probably due to the early processes of talent identification.

## Introduction

To guarantee equal competition, sports governing bodies for youths generally allocate participants to chronological age groups based on a specific cut-off date. As a result, there are age differences among youth of the same age born shortly after the cut-off date relative to those born almost one year after the cut-off date (Vaeyens et al., 2005). In strength-related sports, early born individuals within an age group are more frequently represented (Cobley et al., 2009; Delorme et al., 2010a; Raschner et al., 2012; Till et al., 2010). This is known as the “relative age effect” (RAE) and might have its origin in the selection process, where evaluators mistakenly grant fewer opportunities to athletes born late in a selection period because of their physical disadvantages. This could lead to psychological disadvantages, less instruction, less experience or even less time of play during official games (Díaz del Campo et al., 2010; Mujika et al., 2009; Romann and Fuchslocher, 2011; Vaeyens et al., 2005).

Most of the studies related to the RAE are focused on male sports and it seems to be a persistent and repeated problem both in youth and in senior soccer categories (Cobley et al., 2008; Díaz del Campo et al., 2010; Musch and Grondin, 2011; Pérez-Jiménez and Pain, 2008). However, only a few of the studies examined RAE in female athletes. Furthermore, it was generally assumed that the magnitude of the effect in women’s sports was smaller due to a less intense competition at early ages to reach a position (Delorme et al., 2010a; Musch and Grondin, 2011; Schorer et al., 2009; Till et al., 2010). Moreover, Raschner et al. (2012) suggested that female sports disciplines are not as strength-related as the male variants, and as a consequence, the maturation-related developmental lead is not as decisive.

The presence of RAE in female soccer is still unclear since authors have revealed inconsistent results. For instance, Delorme et al. (2010a) found a significant RAE in all levels of female French players with a classical RAE in youth categories and a different pattern of asymmetry in professional players. On the other hand, Vincent and Glamser (2006) only observed marginal RAEs for girls at a regional level and no RAEs for those playing at a state level in the US competition. Romann and Fuchslocher (2011) showed significant RAEs in the 10 to 14 year-old age group of Swiss female players, but not in the 15 to 20-year-old group. Finally, in a study that analyzed a sample of male and female national youth selections, Helsen et al. (2000) stated that asymmetries in the birth-date distributions of female soccer players were less apparent than in male players.

**Figure 1:**
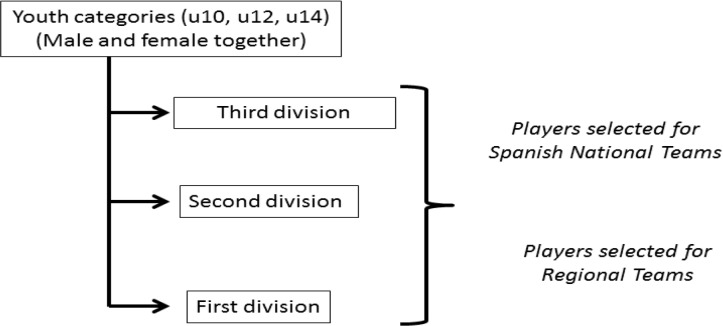
Spanish female soccer structure

Certain sport structures, for instance those in which talented athletes are identified, selected, and developed from a young age, could benefit from inequalities if the number of people wishing to participate grossly exceeds the infrastructure available (Cobley et al., 2008). This could be the case of female soccer in Spain since it currently has a relatively unstructured environment for youth participation in which female and male players can play together until fourteen years of age, when female exclusive competition starts (first, second, and regional division) (Figure 1). At this time, available positions in Spanish elite clubs are limited, whereas low level teams might support universal enrolment. This specific situation could cause a different impact of RAE depending on the competitive level (Delorme et al., 2010b; Diaz del Campo et al., 2010)

Moreover, if RAE is related to physical advantages, it could be assumed that the magnitude of this phenomenon varies according to the players’ position, with stronger RAEs at more physically demanding positions (Schorer et al., 2009; Till et al., 2010). To our knowledge, there are only a few studies related to the influence of a soccer playing position on RAE. In female soccer, several authors pointed out that defenders and goalkeepers showed significantly higher RAEs compared to midfielders, which they linked to physical advantages in these more demanding positions (Baker et al., 2009; Romann and Fuchslocher, 2011).

Because of the lack of related studies and the presence of unclear results, the main purposes of the present study were to examine RAE in the overall setting of Spanish female soccer and to identify if playing positions modified the prevalence and size of RAEs. More specifically, we hypothesized that the present developmental structure of female soccer, with early talent identification procedures, could stimulate the appearance of RAEs at the lowest level of selection and would be even more pronounced in higher competition levels, which could lead to a reduction of the overall quality of these levels. On the other hand, we also hypothesized that the RAE varied according to the players’ position with stronger RAEs in positions with higher physical demands.

## Material and Methods

The study was conducted in accordance with the Declaration of Helsinki and approved by the Ethical Committee of the European University Miguel de Cervantes.

### Participants

The study comprised a total of 4,035 female soccer players that had played for teams registered in competitions organized by the Spanish Royal Federation of Soccer (SRFS) for the 2010–2013 seasons. They were allocated to one of the five subgroups, which included the total amount of players registered in each competitive level.

- First division group (FDG) (n=936)
- Second division group (SDG) (n=1711)
- Third division group (TDG) (n=870) (Castilla y León regional group).
- National team (NT) (n=232): Players selected for the national teams (U17, U19, U21 and senior).
- Regional team (RT) (n=286): Players selected for Castilla y León regional teams (U17 and U19).

Only players born after 1980 were included in the study (Vaeyens et al., 2005).

### Procedures

All data were acquired from the SRFS database. Player’s birth-dates were categorized into quartiles bearing in mind that the selection year corresponds with the regular calendar year. Additionally, the playing position for players in NT, RT, FDG and SDG were registered. The playing position data from TDG players were not available. Table 1 shows the descriptions and distributions of players’ positions used in the analysis.

Expected birth date distributions were calculated for the available Spanish female population birth statistics between 1980 and 1999 (Source: Spanish National Institute of Statistics, Madrid).

### Statistical analysis

Differences between the observed and expected birth-date distributions were tested with the chi-square statistic. Significant chi-square values were followed up by calculating odds ratios and 95% confidence intervals (CI) for the quartile and half-year distributions in order to examine subgroup differences with respect to the bias of the birth-date distribution. The odds ratios compared the birth-date distribution of a particular quartile (Q1, Q2 or Q3) or semester (S1) with a reference group, who consisted of the relatively youngest players (Q4 or S2). A higher odds ratio indicated an increased risk of players who were born in that particular quartile compared to the reference quartile. The level of significance was set at p<0.05.

## Results

**Figure 2:**
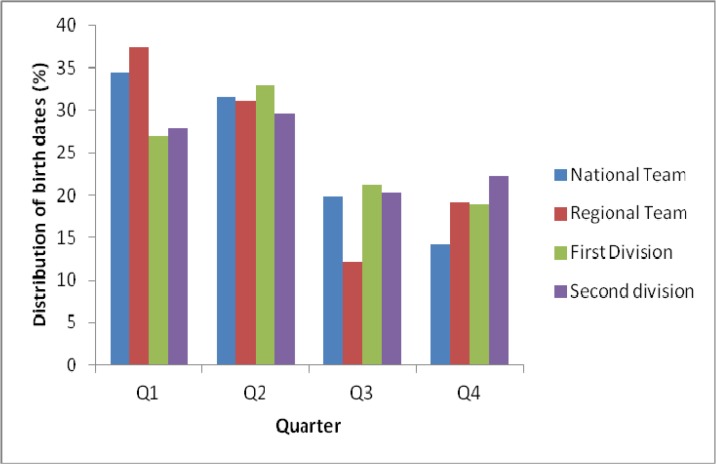
Quarterly distribution of birth dates for the four subgroups of players in which the separate chi-square analyses revealed that the distribution differed significantly from that of the general population. (p<0.05)

Table 2 shows the distributions of births by quarter for the five subgroups and the Spanish general population. Separate chi-square analyses revealed that the birth-date distributions of almost all groups of football players differed significantly from that of the general population. Q1 and Q2 are generally over-represented (Figure 2). Only the birth-date distribution in TDG is not significantly different from that of the general population.

Table 3 displays the results of the odds ratios and the 95% CI. This analysis revealed significant odds ratios when comparing the observed and the expected birth-date distributions. Between-group comparison revealed that the risk was progressively increased with a higher level of involvement. The Q1 vs Q4 odds ratio was the highest in NT, RT, FDG and SDG followed by the Q2 vs Q4 odds ratio. For TDG odds ratios did not show the same pattern.

**Figure 3:**
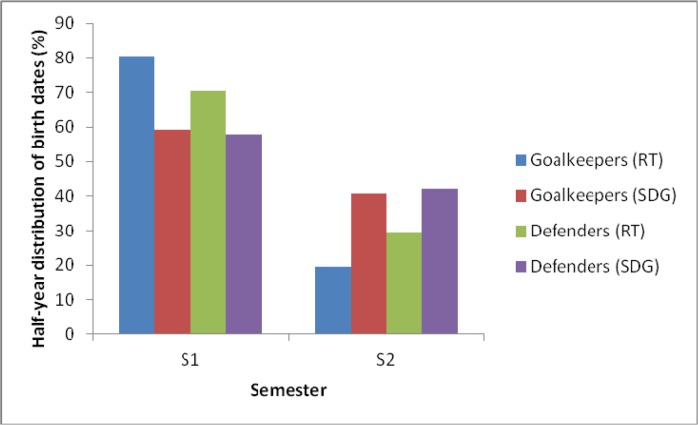
Half-year distribution of birth dates in the subgroups in which the separate chi-square analyses revealed that the distribution differed significantly from the observed birth-date distribution in that level (p<0.05)

Table 4 shows the distribution of birth-dates by semester by playing positions for the four subgroups of Spanish female soccer players. Separate chi-square analyses revealed that in some playing positions birth-date distributions differed significantly from the observed birth-date distribution in that level. That is the case for goalkeepers and defenders in RT and SDG (Figure 3). Quarterly results were not significant for any of the subgroups.

## Discussion

This study examined the relative age effect in the overall setting of Spanish women’s soccer. As predicted, the main findings confirm the existence of a RAE at almost all competitive levels of female soccer with an overrepresentation of players born in the first quartile of the year. Moreover, findings revealed that the RAE was more pronounced as the level of competition increased.

The RAE is a persistent problem in male team sports (Delorme and Raspaud, 2009; Till et al., 2010) and seems to be worldwide in male soccer (Cobley et al., 2008; Mujika et al., 2009; Pérez-Jiménez and Pain, 2008; Simmons and Paull, 2001; Williams, 2010). RAEs have also been found in female athletes, but results are still unclear (Baker et al., 2009; Baxter-Jones, 1995; Delorme and Raspaud, 2009; Helsen et al., 2005; Raschner et al., 2012). For instance, several authors did not find a RAE in different female cohorts of players, elite Swiss soccer teams (Romann and Fuchslocher, 2011) and state team players of the Olympic Development Program in the U.S. (Vincent and Glamser, 2006). Nevertheless, results in the current study are in line with those obtained in other team sports (Delorme and Raspaud, 2009; Weir et al., 2010) and in a sample of French female soccer players of all competitive levels (Delorme et al., 2010a). In contrast to our results, several authors concluded that in female sports, more precisely German handball, US soccer and French soccer, the higher the level the lower the RAE (Baker et al., 2009; Delorme et al., 2010a; Schorer et al., 2009). Similarly, Till et al. (2010) stated that the RAE risk was inflated when representative levels of competition occur.

Along the same line, Pérez-Jiménez and Pain (2008) pointed out that results obtained in national and regional teams could be a good portrait of how selection processes operated at a grassroot level, since there was a selection within the selection and the selection criteria could be again biased.

The main cause of RAE in soccer might be attributed to the talent identification processes (Díaz del Campo et al., 2010; Wattie et al., 2008). Female soccer structure in Spain could stimulate the RAE due to an early selection to form the lower teams of elite clubs. In that context, females who mature early have athletic performance advantages (Malina, 2004), something that could mistakenly lead evaluators to select players based on biological and physical maturity in order to obtain immediate performance success (Cobley et al., 2009; Malina, 2004; Mujika et al., 2009; Wattie et al., 2008). Moreover, selected players could benefit from greater quality coaching and experience at advanced competitive levels (Cobley et al., 2008; Díaz del Campo et al., 2010; Romann and Fuchslocher, 2011; Vaeyens et al., 2005) while the rest of the players would normally remain at a lower performance level of involvement. As late maturing players have less opportunities of being selected for elite teams, they ultimately have a greater likelihood of dropping out of the sport (Delorme et al., 2010b; Musch, 1999; Vaeyens et al., 2005; Williams, 2010). Consequently, many promising talents are likely to be overlooked because of a relative age disadvantage (Musch and Grondin, 2011) while early maturing individuals are not required to develop technical or tactical skills, due to the over-emphasized importance given to physical attributes (Pérez-Jiménez and Pain, 2008; Williams, 2010). Consequently, the overall quality of the highest competitive levels could be reduced due to a waste of potential (Helsen et al., 2000; Musch and Grondin, 2011; Pérez-Jiménez and Pain, 2008; Romann and Fuchslocher, 2011; Simmons and Paull, 2001; Vaeyens et al., 2005). Nonetheless, athletes who mature late may develop technical and decision making skills at an adult age, resulting in a larger repertoire of skills. However, the initial small performance disadvantage in late maturing individuals with respect to early maturing individuals can lead to a large disadvantage that often cannot be compensated for (Ford and Williams, 2011; Mujika et al., 2009; Musch and Grondin, 2011; Schorer et al., 2009;) This could be the case in Spanish female soccer where the RAE is maintained at the highest levels.

A RAE was not found at the lowest level. This could be related to the so called “depth of competition hypothesis”, since that level might support universal enrollment and there is no great competition for limited posts on a team (Baxter-Jones, 1995; Cobley et al., 2009; Delorme et al., 2010b; Musch and Grondin, 2011).

On the other hand, as predicted, results revealed that the birth-date distributions at specific levels, in some playing positions, were significantly biased towards a higher number of births during the early part of the selection. Di Salvo and Pigozzi (1998) stated that goalkeepers and central defenders were more physically demanding positions and, because of that, results in male and female players showed that the presence of the RAE might in part be position dependent (Romann and Fuchslocher, 2011; Schorer et al., 2009; Till et al., 2010; Weir et al., 2010). Evaluating our results, it could be speculated that coaches at some competitive levels of women’s soccer may also tend to select relatively older defenders and goalkeepers because of the physical maturity.

To our knowledge, no strategies have been implemented to combat the negative consequences of the RAE in female soccer. However, as mentioned before, a decrease in RAEs might substantially enhance the overall quality of female soccer. Several alternatives have been proposed to reduce the impact of the RAE: rotating cutoff dates or grouping according to physical characteristics (Brewer et al., 1995; Cobley et al., 2009; Musch and Grondin, 2011; Romann and Fuchslocher, 2013; Schorer et al., 2009; Simmons and Paull, 2001). However, in our sample the main problem appeared to be the early selection process that exposes players to high-level competition very early. A possible first step could be to change the mentality of team coaches, emphasizing skill assessment when identifying players rather than aspects of performance underpinned by physical attributes (Díaz del Campo et al., 2010; Helsen et al., 2000; Musch and Grondin, 2011; Romann and Fuchslocher, 2013). It has also been proposed that selection processes into elite soccer pathways should be delayed until the late teen years to keep players who are at physical disadvantage involved in the sport until they have fully matured (Cobley et al., 2008; Díaz del Campo et al., 2010; Romann and Fuchslocher, 2011) but it is rather difficult while specialization is growing.

## Conclusions and practical implications

In conclusion, the current study shows the presence of RAE in the present structure of Spanish female soccer. Moreover, RAE is more pronounced as the competitive level increases, which might lead to a reduction of the overall quality of the competition due to waste of talent. Consequently, it is necessary to implement strategies to reduce the problem. One of the first steps could be to change the mentality of the coaches when they select players, finding better balance between short-term success and a more process-oriented approach to instruction. On the other hand, it could be interesting to delay the selection process until late teens, when players reach physical maturity, however, this is rather difficult as specialization starts very early in elite sport. Moreover, coaches should keep in mind that they normally select relatively older defenders and goalkeepers because of physical maturity, what could reduce future progress of a player.

## Figures and Tables

**Table 1:** Description and distribution of players’ positions

Position	NT	RT	FDG	SDG	Total
Goalkeeper	22	36	97	177	332
Defender	In this position the database included centre-backs, wing-backs and full backs without distinction				
	66	95	300	552	1013
Midfielders	In this position the database included centre-midfielders, defensive-midfielders, attacking-midfielders and wide-midfielders without distinction				
	60	59	205	333	657
Forwards	In this position the database included centre-forward, second-striker and winger without distinction				
	55	70	207	373	705

**Table 2:** Quarterly distribution of birth dates for the five subgroups and for the Spanish general population

Group	Q1	Q2	Q3	Q4	Total	*X^2^*	*p*
National team	80 (34,5%)	73 (31,5%)	46 (19,8%)	33 (14,2%)	232	25.48	0.0001
Regional team	107 (37,4%)	89 (31,1%)	35 (12,2%)	55 (19,2%)	286	44.02	0.0002
First division	252 (26,9%)	308 (32,9%)	199 (21,3%)	177 (18,9%)	936	43.90	0.0001
Second division	477 (27,9%)	506 (29,6%)	348 (20,3%)	380 (22,2%)	1711	40.18	0.0003
Third Division	255 (29,3%)	185 (21,3%)	219 (25,2%)	211 (24.3%)	870	5.25	*0.145*
Spanish population	1048768 (24.6%)	1086980 (25.5%)	1079649 (25.4%)	1043592 (24.5%)	4258989	1.04	*0.254*

**Table 3:** Odds ratios (and the 95% confidence interval) examining birth-date distributions in relation to female soccer players’ subgroups

	Odds ratio comparisons (95% confidence interval)			
Group	Q1 vs Q4	Q2 vs Q4	Q3 vs Q4	S1 vs S2
National team	2.68 (1.99–4.32)	2.60 (2.30–5.03)	1.09 (0.88–2.72)	2.18 (2.02–3.45)
Regional team	2.24 (2.34–5.51)	2.15 (2.22–4.75)	0.75 (0.71–1.27)	2.10 (2.13–3.99)
First division	2.02 (2.15–4.01)	2.02 (2.28–4.15)	0.99 (0.86–0.98)	2.07 (1.99–3.07)
Second division	1.22 (0.97–1.83)	1.03 (0.93–1.54)	0.89 (0.88–1.07)	1.54 (1.75–2.72)
Third Division	0.66 (1.07–1.11)	0.74 (1.02–1.11)	0.43 (0.75–0.85)	0.32 (1.09–1.17)

**Table 4:** Half-year distribution of birth dates for the five subgroups of Spanish female soccer players

Competitive level	Position	S1	S2	Total	X^2^	p
Goalkeepers (NT)		13 (59.1%)	9 (40.9%)	22	0.468	0.494
Goalkeepers (RT)		29 (80.5%)	7 (19.4%)	36	6.003	0.038
Goalkeepers (FDG)		60 (61.9%)	37 (38.1%)	97	0.139	0.709
Goalkeepers (SDG)		105 (59.3%)	72 (40.7%)	177	9.231	0.022
Defenders (NT)		41 (62.1%)	25 (37.9%)	66	0.442	0.506
Defenders (RT)		67 (70.5%)	28 (29.5%)	95	8.571	0.035
Defenders (FDG)		180 (60%)	120 (40%)	300	0.001	0.998
Defenders (SDG)		319 (57.8%)	233 (42.2%)	552	8.031	0.021
Midfielders (NT)		60 (67.4%)	29 (32.6%)	89	3.416	0.065
Midfielders (RT)		59 (69.4%)	26 (30.6%)	85	0.007	0.935
Midfielders (FDG)		205 (61.7%)	127 (38.2%)	332	0.422	0.516
Midfielders (SDG)		333 (56.3%)	258 (43.6%)	591	0.189	0.604
Forwards (NT)		30 (54.5%)	25 (45.5%)	55	2.498	0.111
Forwards (RT)		47 (67.1%)	23 (32.8%)	70	0.914	0.339
Forwards (FDG)		112 (54.1%)	95 (45.9%)	207	3.777	0.092
Forwards (SDG)		201 (53.9%)	172 (46.1%)	373	2.590	0.108

The expected birth-date distribution corresponds to the observed birth-date distribution in each competitive level
